# Association of Serum Vitamin D With Psoriasis and Effect Modification by Central Obesity

**DOI:** 10.3389/fmed.2020.00236

**Published:** 2020-06-18

**Authors:** Yehong Kuang, Yi Xiao, Zhiqin Fang, Yichi Zhang, Minxue Shen, Xiang Chen, Mingliang Chen, Chengzhi Lv, Wu Zhu

**Affiliations:** ^1^Department of Dermatology, Xiangya Hospital, Central South University, Changsha, China; ^2^Hunan Engineering Research Center of Skin Health and Disease, Central South University, Changsha, China; ^3^Hunan Key Laboratory of Skin Cancer and Psoriasis, Central South University, Changsha, China; ^4^The First High School of Changsha, Changsha, China; ^5^Dalian Dermatosis Hospital, Dalian, China

**Keywords:** effect modification, obesity, psoriasis, serum vitamin D, waist-hip ratio

## Abstract

**Background:** Psoriasis is a chronic recurrent inflammatory disease involving many common mechanisms associated with obesity, such as systemic inflammation and vitamin D deficiency. This study aimed to examine the association of the serum concentration of 25-hydroxyvitamin D with psoriasis and the effect modification by obesity among the affected patients.

**Methods:** A mixed cross-section study was conducted. We consecutively included untreated psoriasis patients from the outpatients who visited the Department of Dermatology of Xiangya Hospital and recruited 205 gender-matched healthy controls from the Hunan Civil Servant Cohort. In both groups, we measured the serum 25-hydroxyvitamin D level, body mass index (BMI), waist-hip-ratio (WHR) and other psoriasis-related clinical indicators.

**Results:** A total of 203 psoriasis outpatients and 205 gender-matched cohort participants with complete data of serum vitamin D concentration were included in the analysis. The serum vitamin D levels of the two groups were close to each other, while the mean WHR of the psoriasis outpatients was significantly higher. Compared with the controls, the risk of psoriasis increased significantly when the vitamin D level decreased from 20 to 10 nmol/L. A significant interaction between the serum vitamin D level and the obesity category (BMI × WHR) was identified. After stratification by WHR, vitamin D was not associated with psoriasis in subjects with normal WHR. In contrast, the association between vitamin D deficiency and psoriasis retained and the effect size augmented in patients with central obesity.

**Conclusions:** WHR may modify the association between serum vitamin D and psoriasis. Treatment advocating Vitamin D supplements may tailor to psoriasis patients with metabolic disorders.

## Introduction

Psoriasis is not only a T-helper-1 (Th1)/Th17-mediated chronic inflammatory skin disease (1) but also a systemic disease involving higher risks of metabolic disorders such as obesity (2), and other metabolic-related diseases. Vitamin D plays a vital role in both bone health and non-skeletal diseases. In addition, vitamin D insufficiency has been reported in numerous major non-communicable diseases (3), such as diabetes (4), cardiovascular diseases (5), obesity (6), and other metabolic disorders. Meanwhile, there were sporadic hospital-based studies showing that the serum vitamin D concentration also decreased in psoriasis patients (7–13) based on relatively small samples.

The 25-Hydroxy vitamin D [25(OH)D] is currently considered the most accurate biomarker of the serum vitamin D level. It is derived from the daily dietary intake (14) and the cutaneous synthesis. A majority of researchers have agreed that 25(OH)D level <20 ng/mL can be used to define vitamin D insufficiency or even deficiency (15), which may implicate adverse health effects. Among numerous factors, the role of obesity in decreasing the serum 25(OH)D level (16) has been well-acknowledged (17). However, to our best knowledge, no study has been designed to evaluate the relationship between obesity and the serum concentration of 25(OH)D in patients with psoriasis. It is worth comparing the vitamin D status between patients with psoriasis and the control population stratified by obesity as measured by BMI and WHR. Thus, we conducted a case-control study to uncover the association of vitamin D with psoriasis and to examine the effect modification by obesity.

## Materials and Methods

### Study Participants

This is a case-control study targeting at a population comprised of psoriasis outpatients from the Department of Dermatology of Xiangya Hospital. Demographic information was collected after the recruitment of subjects.

This study was conducted according to the guidelines established in the Declaration of Helsinki. All patients-involved procedures had been approved by the institutional research ethics board of Xiangya Hospital and Xiangya School of Public Health, Central South University, Changsha, China (approval number: XYGW-2016-10). Informed consent had been obtained from all subjects before the investigation. All the recruited patients were diagnosed of psoriasis by an associate professor or above, and had not received any treatment. The main target range of age was 18–70 years. The control group was comprised of the participants from the Hunan Civil Servant Cohort. An even distribution of sex and age was ensured by stratified random sampling from the cohort participants.

Participants who had acute or chronic infection, digestive diseases, kidney diseases, metabolic and nutritional diseases, rheumatic diseases, endocrine diseases, or circulation system diseases, and who had undergone surgery or received medication such as vitamin supplements, blood donation or transfusion within the previous 6 months were excluded.

### Exposure Assessment

The serum 25(OH)D level was measured by radioimmunoassay (RIA) (18, 19). Briefly, 5 ml of serum sample was drawn from the whole blood sample obtained from the antecubital vein of the participant to be tested. The process was operated by a qualified phlebotomist in the morning under a specified centrifugal force (2,500 rpm for 10 min at room temperature). Within 2 h, the serum sample would be then collected and stored in a refrigerator at −80°C until further detection. A commercial ^125^I-25(OH)D RIA kit (DiaSorin, Stillwater, Minnesota, USA) was used to analyze the serum 25(OH)D level in the same laboratory.

### Outcome Assessment

Psoriasis outpatients were consecutively recruited from the dermatology clinic. Psoriasis in the cohort participants was diagnosed by certified dermatologists during field survey and the diagnosis results have been added in the final analysis. The age- and sex-matched healthy controls were randomly selected from the cohort participants.

### Assessment of Confounders

Height, weight, waist circumference, and hip circumference were measured by a research nurse using standardized methods. BMI was calculated as weight (kg)/height^2^ (m^2^). WHR was calculated as waist circumferences (cm)/hip circumference (cm). BMI was categorized by the cut-off of 24 kg/m^2^ (20), and WHR was categorized by the cut-off of 0.85 for females and 0.9 for males (21).

### Statistical Analyses

Continuous data was presented as the mean ± standard deviation, and the between-group differences were tested using analysis of variance (ANOVA). Categorical data was presented as percentage (%), and the between-group differences were tested using the chi-square test. Generalized additive models were used to explore the association of vitamin D with psoriasis. Because U-shape associations were identified, quadratic regression models were further used for parametric estimation. Interactions between vitamin D and obesity indices were detected, and stratification analyses by obesity category were performed for any significant interactions. A *P* < 0.05 was considered statistically significant. Analyses were performed using SAS software version 9.4 (SAS Institute, Inc., Cary, North Carolina, United States).

## Results

A total of 203 psoriasis outpatients and 205 gender-matched cohort participants with complete data of serum vitamin D concentration were included in our analysis. Two subjects with psoriasis identified in the cohort were also included (the prevalence of psoriasis among the cohort participants was 0.98%). The serum vitamin D levels of the two groups were close to each other, while the mean WHR of the psoriasis outpatients was significantly higher (Table 1).

**Table 1:** Characteristics of participants.

**Characteristics**	**Cohort participants (*n* = 205)**	**Psoriasis outpatients (*n* = 203)**
Age, *y* (mean ± SD)	46.0 ± 9.3	41.4 ± 15.3
Female (*n*, %)	60 (29.3)	61 (30.0)
BMI, kg/m^2^ (mean ± SD)	24.3 ± 3.1	23.1 ± 3.3
BMI ≥ 24 kg/m^2^ (*n*, %)	108 (52.7)	79 (38.9)
WHR (mean ± SD)	0.87 ± 0.06	0.90 ± 0.07
Central obesity (*n*, %)	74 (36.1)	126 (62.1)
Vitamin D, nmol/L (mean ± SD)	23.6 ± 7.7	24.0 ± 9.2
Vitamin D <25 nmol/L (*n*, %)	131 (63.9)	115 (56.6)
Psoriasis (*n*, %)	2 (1.0)	203 (100.0)

*BMI, body mass index; WHR, waist-hip ratio; SD, standard deviation*.

**Figure 1:**
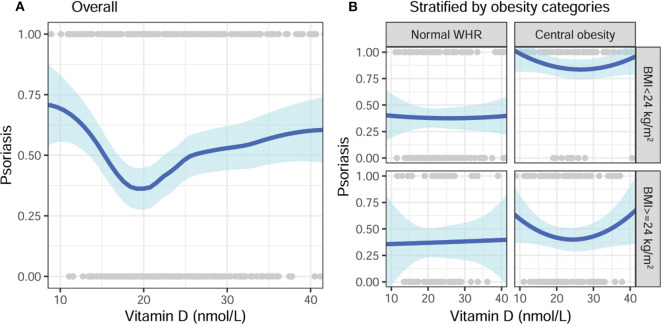
Unadjusted association of vitamin D with psoriasis, stratified by obesity categories. **(A)** Overall and **(B)** stratified by BMI and WHR categories. BMI, body mass index; WHR, waist-hip ratio.

The vitamin D level showed a non-linear U-shape association with psoriasis according to the generalized additive model (Figure 1A). The risk of psoriasis significantly decreased when the vitamin D level increased from 10 to 20 nmol/L, and then slightly increased after that. Because significant interactions between the vitamin D level and obesity category (BMI × WHR) were identified, stratification analyses were then performed. As shown in Figure 1B, vitamin D was not associated with psoriasis in subjects with normal WHR despite the BMI category. In contrast, the association exhibited a U-shape in those with central obesity (large WHR) despite the BMI category.

**Figure 2:**
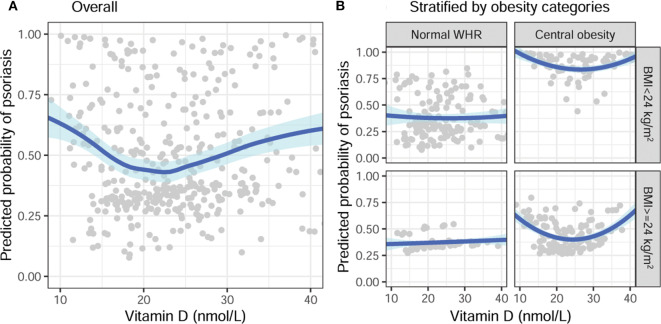
Adjusted association of vitamin D with a predicted probability of psoriasis, stratified by obesity categories, adjusted for age and gender. **(A)** Overall and **(B)** stratified by BMI and WHR categories. BMI, body mass index; WHR, waist-hip ratio.

After fitting data with quadratic regression, the parametric estimation of the effect size of vitamin D is shown in Table 2. With stratification by obesity category, vitamin D was not significantly associated with psoriasis in subjects with normal WHR. Consistently, the quadratic terms of vitamin D were marginally significant in subjects with central obesity, despite the BMI category. After adjustments for age and gender, the associations remained consistent, although some were no longer statistically significant (Figure 2A). In subjects with central obesity, the risk of psoriasis was the lowest when the serum 25(OH)D was around 25 nmol/L (Figure 2B).

**Table 2:** Effect modification of association of vitamin D with psoriasis by obesity.

	**Basic model**		**Adjusted model^a^**	
**Stratum**	**β (SE)**	***P***	**β (SE)**	***P***
Overall (*n* = 408)				
VitD	−0.095 (0.053)	0.0745	−0.096 (0.055)	0.0790
VitD quadratic term	0.002 (0.001)	0.0573	0.002 (0.001)	0.0415
BMI <24 kg/m^2^ and normal WHR (*n* = 141)				
VitD	−0.020 (0.072)	0.7820	−0.037 (0.080)	0.6434
VitD quadratic term	0.000 (0.001)	0.7523	0.001 (0.001)	0.4413
BMI ≥ 24 kg/m^2^ and normal WHR (*n* = 67)				
VitD	0.004 (0.167)	0.9799	0.061 (0.178)	0.7309
VitD quadratic term	0.000 (0.003)	0.9939	−0.001 (0.003)	0.7699
BMI <24 kg/m^2^ and central obesity (*n* = 80)				
VitD	−0.548 (0.358)	0.1261	−0.502 (0.362)	0.1654
VitD quadratic term	0.011 (0.007)	0.1309	0.010 (0.007)	0.1417
BMI ≥ 24 kg/m^2^ and central obesity (*n* = 120)				
VitD	−0.207 (0.118)	0.0796	−0.191 (0.112)	0.0893
VitD quadratic term	0.004 (0.002)	0.0734	0.004 (0.002)	0.0576

*SE, standard error; vitD, vitamin D; BMI, body mass index; WHR, waist-hip ratio*.

a *An Adjusted for age and gender*.

## Discussions

Our study showed that the association between serum vitamin D and psoriasis might be modified by central obesity, indicated by WHR. Inconsistent with the findings in the majority of previous studies (7, 22), our results showed that there was no significant difference in serum vitamin D level between the psoriasis patients and healthy controls. However, a significant interaction between the serum vitamin D level and obesity was identified. This is the first time that the serum vitamin D level in Chinese psoriasis patients was reported. Our results also showed that, interestingly, serum 25(OH)D deficiency was associated with a higher risk of psoriasis only in the subgroup of abnormal WHR. This inconsistent finding suggested that the impact of decreased vitamin D on psoriasis pathogenesis might be related to central obesity, a well-acknowledged comorbidity of psoriasis.

Unfortunately, no previous study has explored the modification effects of WHR on the association between serum vitamin D and psoriasis. Nevertheless, vitamin D deficiency has been multi-dimensionally confirmed to correlate with obesity (6, 23–26), especially central obesity. The reported relationship between psoriasis and decreased serum Vitamin D may be mediated by the shared mechanism of the coexistence of psoriasis and central obesity (27).

Another explanation for our result lies in the specialty for the studied population. After a long time of poverty, the Chinese population has been increasingly experiencing a non-communicable tsunami, especially for central obesity (28) and diabetes (29), since the opening-and-reform-up from the 1980s. Within the past four decades, the number of psoriasis patients has been significantly increased (30), and the epidemic of psoriasis has shifted from a genetic-dominated to a comorbidity-driven epidemic in China. The climbing prevalence of central obesity among Chinese psoriasis patients might help us distinguish the relationship among psoriasis, central obesity, and serum vitamin D. Besides, the average serum vitamin D level in the healthy Chinese population has been reported to be lower than that in the western population (31, 32). A multi-center research in 2013 showed that the average serum vitamin D level (31) in the general Chinese population was around 25 nmol/L, lower than the reported average according to the western standard (33). The fact that vitamin D insufficiency in the Chinese population is too common to show any difference between the psoriasis patients without central obesity and the healthy controls might be another explanation for the inconsistent finding.

One limitation of this study is selection bias. The healthy controls were recruited from an ongoing cohort, and the participants who received vitamin D test were mostly the elderly people (generally >40 years). Another significant limitation of our study is that the participants were recruited across a 1-year period, which cannot erase the confounding effect from the seasonal change of serum vitamin D status owing to the difference in exposure to sunlight (34). However, all participants in our study resided in Hunan province, and the difference due to latitude can be ignored.

## Data Availability Statement

All datasets generated for this study are included in the article/supplementary material.

## Ethics Statement

Ethical review and approval was not required for the study on human participants in accordance with the local legislation and institutional requirements. The patients/participants provided their written informed consent to participate in this study.

## Author Contributions

YK and YX performed study design, data analysis, and manuscript writing. YZ, ZF, XC, MC, and MS contributed to data collection and validation. MC and XC performed clinical diagnosis and samples collection. CL and WZ: clinical experts and manuscript revision. All authors read and approved the final version of the manuscript.

## Conflict of Interest

The authors declare that the research was conducted in the absence of any commercial or financial relationships that could be construed as a potential conflict of interest.
